# Numerosity Perception and Perceptual Load: Exploring Sex Differences Through Eye-Tracking

**DOI:** 10.3390/jemr18020009

**Published:** 2025-04-03

**Authors:** Julia Bend, Anssi Öörni

**Affiliations:** Faculty of Social Sciences, Business and Economics, and Law, Åbo Akademi University, 20500 Turku, Finland

**Keywords:** eye-tracking, attention, individual differences, numerosity perception, perceptual load, sex differences, visuospatial abilities, eye movements

## Abstract

This study investigates sex differences in numerosity perception and visuospatial abilities in adults using eye-tracking methodology. We report the results of a controlled dual-task experiment that assessed the participants’ visuospatial and numerosity estimation abilities. We did not observe sex differences in reaction times and accuracy. However, we found that females consistently underestimated numerosity. This underestimation correlated with higher perceptual load in females, as evidenced by shorter fixation durations and increased fixation rates. These findings suggest that perceptual load, rather than visual or spatial abilities, significantly influences numerosity estimation. Our study contributes novel insights into sex differences in both numerosity estimation and visuospatial abilities. These results provide a foundation for future research on numerosity perception across various populations and contexts, with implications for educational strategies and cognitive training programs.

## 1. Introduction

What causes the sex difference in numerosity perception is one of the unanswered questions in numerosity research. Numerosity perception, the spontaneous extraction and mental representation of set size, is a fundamental ability that significantly influences human behavior [1]. We use numerosity perception to judge quantity or probability, often relying on numbers rather than other available types of information. Understanding the principles of numerosity perception is important: Numerosity perception is not only a life skill with survival value but is also vital in today’s world because it is closely related to mathematical abilities [2].

Numerosity perception, as a fundamental cognitive ability, is universal and emerges regardless of cultural experience or development [3]. That we all have the skill, as do many other complex animal species, does not mean that we are all equally good at numerosity perception. Krueger [4] demonstrated that several systematic differences exist in the accuracy of numerosity estimation. One of the more intriguing differences was that, compared to males, females tend to underestimate numerosity. Krueger speculated that this could be due to potential differences in visual acuity, visuospatial processing, and mathematical skills. Although Krueger’s [4] observation of sex differences in numerosity perception provided an early benchmark, subsequent replication of this specific finding has been limited, underscoring the need for further investigation into its underlying mechanisms.

In this paper, we seek to identify what causes the previously observed sex difference in numerosity perception. To test for potential sex differences in both visuospatial abilities and numerosity perception in a single experiment, we designed a dual-task experiment with two distinct but interrelated tasks: an attention-demanding multitarget visual search and a numerosity estimation task.

### 1.1. Previous Explanations for Sex Differences in Numerosity Perception

Krueger [4] observed that individuals tend to estimate numerosity accurately or overestimate it until the number of elements reaches a threshold of 10 to 20 items, beyond which they generally begin to underestimate. He also found a pronounced tendency among females to underestimate numerosity compared to males, with women reporting lower numerosity estimates at nearly all stimulus levels.

Krueger proposed several potential explanations for these sex differences in numerosity perception [4]: Firstly, he pointed to differences in visual acuity, which tends to be better in males, especially under well-lit conditions, allowing them to see objects more clearly and estimate numerosity more accurately. Secondly, he considered differences in arithmetic reasoning and quantitative evaluations, suggesting that differences in how the sexes approach magnitude estimation and production could play a role in numerosity estimation. Thirdly, Krueger discussed visual–spatial abilities. Historically, sex differences in visual-spatial ability have been considered among the most persistent individual differences in multifactor psychological tests. Males have decidedly better spatial skill than females [5]. Even if more recent studies report smaller differences in spatial ability, the differences remain non-trivial [6,7,8].

### 1.2. Selective Attention as a Cause of Sex Difference in Numerosity Perception

One possible explanation for the observed sex difference in numerosity perception is the difference in the ability to inhibit distracting information. Inhibition of responses is one of the few cognitive functions where a consistent sex difference is observed, as supported by research indicating that women exhibit better behavioral inhibitory control than men, demonstrated by faster detection and earlier resolution of response conflicts [9]. Numerosity perception involves searching for and identifying objects that belong to a group. In natural environments this means, among other things, distinguishing candidate targets from distractors. Selective attention preferentially processes relevant sensory information and filters out distracting information [10]. Selective attention depends on an active inhibition mechanism, which activates following the initial parallel processing of both target and distractor information [11]. Inhibition suppresses cognitive processing that is irrelevant to the task [12]. Neurophysiological observations suggest that most of the brain’s electrophysiological activity is specifically related to inhibition [13].

Although recent findings suggest that some of the differences in sustained attentional control, such as omission and commission errors, are related to sociocultural factors, the sex difference in reaction time appears to persist even after controlling for sociocultural influence [14]. The difference in inhibitory control is not limited to selective attention: Men are significantly more prone than women to impulse control disorders [15], for example, impulsive behavior and addictions [16]. The central importance of inhibitory control in producing selective attention, along with the strong sex difference observed in inhibitory control, suggests that differences in numerosity perception may also be influenced by sex-related differences in inhibition. Specifically, when exposed to a moderate number of candidate targets (including both task-relevant targets and distractors), women are expected to be more effective at ignoring distractors compared to men. This greater efficiency in distractor suppression may lead to lower estimates of distractor group sizes. Furthermore, more effective inhibition should result in a different distribution of mental load between men and women.

According to the load theory of selective attention [17], distractor processing depends on the interaction between perceptual and working memory load. High perceptual load in task-relevant stimuli exhausts perceptual capacity, leaving no spare capacity for distractor processing, thus enabling early selection. Conversely, low perceptual load leaves spare capacity that spills over to distractor processing, leading to late selection. Additionally, higher working memory load in tasks requiring active cognitive control increases distractor interference by limiting processing priorities.

### 1.3. Current Study

In this publication, we use previously unanalyzed data, which are part of a larger material. We have published results based on other subsets of the material in our previous publications [18,19]. To assess both visuospatial abilities and numerosity perception within a single experiment, we designed a dual-task setup. This included an attention-intensive visual search task paired with a numerosity estimation task. Our goal was to examine sex differences in numerosity estimation in a more natural-like environment. As König et al. [20] highlighted, it is important to study eye movements not only in controlled settings but also in more natural, unrestricted environments. To address this, we incorporated a novel approach by using three-dimensional moving objects rather than the traditionally employed two-dimensional ones. Through this dual-task experiment, we sought to explore whether the sex differences observed in numerosity estimation may stem from functional differences in selective attention, possibly due to variations in the inhibitory mechanism.

## 2. Methods

### 2.1. Participants

Twenty-one participants, twelve females (mean age = 29.2, range = 20–50), partook in the experiment. Each had normal or corrected-to-normal vision. Participation was voluntary, with written consent obtained beforehand. Participant privacy was maintained in accordance with the Declaration of Helsinki. Participants were unaware of the experimental conditions prior to the study.

### 2.2. Materials

In our experiment the participants were shown a simulated 3D street view tour, which included variable numbers of oncoming vehicles, cyclists, and pedestrians. We utilized 3D moving objects to create realistic 3D video simulations, based on data from the KITTI dataset [21]. The KITTI dataset was recorded using a moving vehicle equipped with camera systems, laser scans, GPS/IMU systems, and accelerometers while driving in and around Karlsruhe, Germany. Originally designed to advance computer vision and robotics for autonomous driving, this dataset provided a rich basis for generating stimuli.

**Figure 1:**
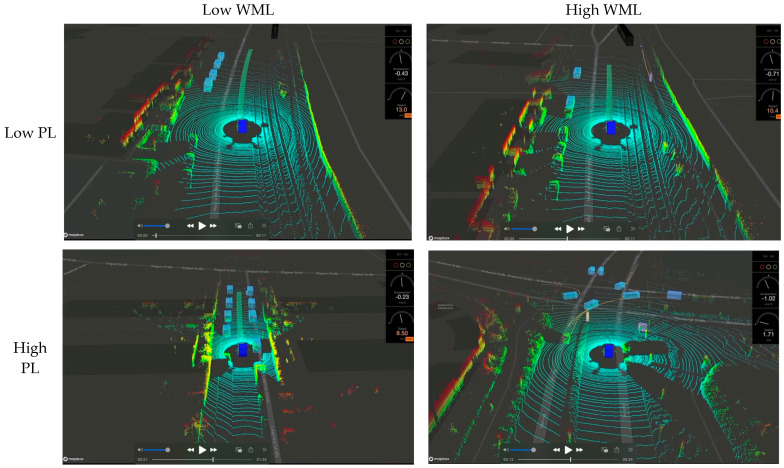
An example of different mental load conditions adopted in the experiment (perceptual load (PL) and working memory load (WML)). The central blue rectangle represents a car from the KITTI dataset, retained for naturalistic context but not counted in the numerosity task. Violet boxes represent bicycle targets, orange boxes represent pedestrian targets, and light blue boxes represent distractors. Participants were instructed to focus on violet and orange targets for identification and ignore the central blue car. To preserve the naturalistic properties of the KITTI dataset, object counts within each load condition varied slightly within predefined thresholds (e.g., 1–9 distractors in low PL—low WML; 1–9 objects, including targets, in low PL—high WML). This variability ensured ecological validity while maintaining experimental control over load categories.

The experimental stimuli were designed to balance experimental control with real-world relevance. These videos were processed using XVIZ v2.0.0 and visualized with streetscape.gl v1.0.0 (both developed by Uber Advanced Technologies Group, San Francisco, CA, USA) within the Mapbox environment (Mapbox GL JS v2.0.0; Mapbox, Inc., Washington, DC, USA). To allow for precise manipulation of target and non-target objects, we replaced the original vehicles, pedestrians, and cyclists with simplified 3D bounding boxes of different colors. For example, pedestrians were consistently represented by orange boxes, while bicycles were represented by pink boxes. Importantly, the speed, movement patterns, and direction of movement of all original objects were preserved, ensuring that the resulting stimuli maintained a naturalistic representation of a driving environment. The final stimuli consisted of 22 short video files, each approximately one minute in length (see Figure 1 for an example). The experiment was programmed with SR Research ExperimentBuilder v2.3.38 (SR Research Ltd., Ottawa, ON, Canada) and controlled using the EyeLink Portable Duo v6.12 system (SR Research Ltd., Ottawa, ON, Canada). Eye movements were recorded with the EyeLink Portable 1000 (SR Research Ltd., Ottawa, ON, Canada), using a head-fixed mount and chest rest to ensure consistent head positioning, viewing distance, and minimized data loss across participants.

To investigate the effects of perceptual and working memory load on participant performance, we systematically manipulated both factors within our task. Perceptual load (PL) was modulated by varying the number of distractor objects (oncoming vehicles) present in the driving scene. Increasing the number of distractors elevated the visual complexity of the display, requiring participants to process a greater amount of visual information. This manipulation primarily affected early perceptual processing. Working memory load (WML) was manipulated by the presence and number of targets (oncoming pedestrians or cyclists). While the presence and number of targets directly influenced the level of working memory load (WML), participants were instructed to remember the features of potential targets throughout the experiment. This requirement to maintain target features in working memory resulted in a baseline level of WML that was present even in trials without visible targets. The presence of targets further increased WML, as participants were required to encode and maintain information about each individual target. Furthermore, participants needed to track which targets they had already responded to, adding a further demand on working memory, particularly as the number of targets increased. Each video contained between zero and four targets, directly impacting the WML. Figure 1 provides an illustration of the four load conditions resulting from this 2 × 2 factorial manipulation (low PL—low WML, low PL—high WML, high PL—low WML, and high PL—high WML). The high PL—high WML condition presented participants with both a high number of distractors and the cognitive challenge of retaining information about multiple targets. Conversely, the low PL—low WML condition minimized perceptual demands (few distractors) while maintaining a baseline level of working memory load (WML) due to the ongoing need to remember target features. To preserve the naturalistic properties of the KITTI dataset, object counts within each load condition varied slightly within predefined thresholds (e.g., 1–9 distractors in low PL—low WML; 1–9 objects, including targets, in low PL—high WML). This variability ensured ecological validity while maintaining experimental control over load categories. The number of trials for each condition was as follows: (1) low PL—low WML: 6 trials; (2) low PL—high WML: 4 trials; (3) high PL—low WML: 2 trials; (4) high PL—high WML: 10 trials.

### 2.3. Procedure

**Figure 2:**
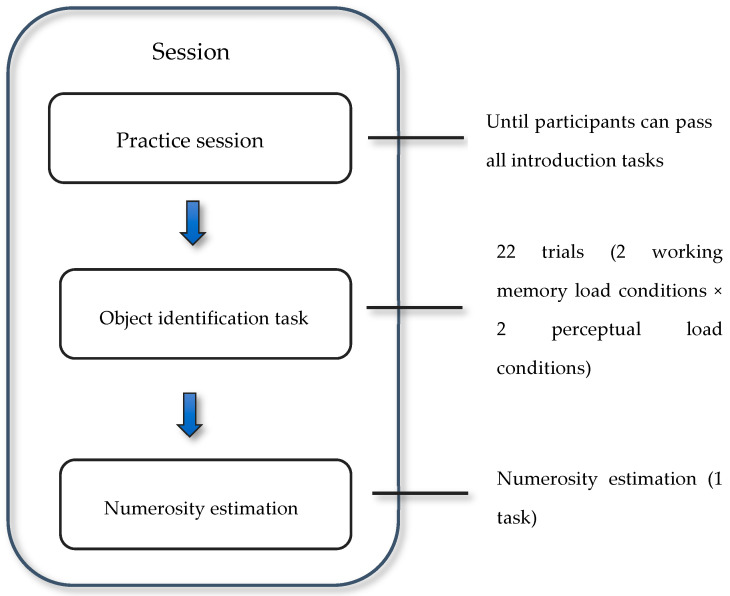
The procedure of one session in the experiment.

The task consisted of two blocks. The first block included two instructional examples, while the second block had twenty-two trials with approximately one-minute video files. All participants viewed the same videos in the second block, featuring varying numbers of targets and irrelevant objects. The number of targets presented at the same time on the screen varied from one to four. Targets appeared in 14 of the 22 videos, while the remaining 8 videos contained only distractors. The number of objects (targets and distractors) in a video ranged from 1 to 17. The procedure of one session in the experiment is shown in Figure 2.

Participants performed two tasks during the experiment: 1. Object Identification Task. Participants were required to press predefined buttons when specific targets (pedestrians or bicycles) appeared in the videos; 2. Numerosity Estimation Task. At the end of the experiment, participants were asked to estimate the number of objects presented in the final trial. Participants received photo instructions on identifying targets before the experiment, without information on the number of objects or visual conditions. They were unaware of the numerosity task to prevent active counting. The entire experiment lasted about 25 min. In designing the numerosity estimation task, we adopted an approach similar to Krueger’s [4] method, which focused on singular judgments to mitigate bias. Krueger argued that avoiding comparative and sequential measurements could yield a more precise and authentic measure of perceived numerosity.

### 2.4. Measurements

Various signals have been identified as valid indicators of an individual’s mental load, such as cardiovascular signals, brain activities, and eye movements [22,23,24,25]. Notably, eye movements are particularly effective at capturing changes in mental load corresponding to the visual demands of a task [24].

We use fixation-related parameters, rather than pupil size, to measure mental load. A fixation is commonly defined as the period between the end of one saccade and the start of the next, during which the eyes remain relatively still, and it is a commonly used measure in eye-tracking research [26]. During active scene perception, our eyes move via saccadic movements, fixating on objects and elements for varying times. Variability in fixation duration is influenced by attentional, perceptual, and cognitive processes involved in scene analysis and comprehension [27]. Fixation duration is a reliable predictor of task performance, correlating with high load [28,29]. While pupil size has shown a positive correlation with mental load across various scenarios [30,31,32], it cannot differentiate between perceptual and working memory load.

Fixation rate—the number of fixations per second or minute—is negatively correlated with task difficulty [33]. Although it varies with task performance, it can serve as a measure of mental load [28]. However, while fixation-related parameters are valuable, there is still some ongoing discussion regarding their effectiveness for assessing mental load [22,30,31,32]. Studies have shown conflicting results, with some finding increased fixation durations associated with higher load [31,32], while others found decreased durations [30]. Tao et al. [24] hypothesize that these inconsistencies arise from differences in the types of mental load assessed. Each task imposes different levels of perceptual load (early processing filter) or working memory load (late processing filter), affecting what and how many items can be processed. Load theory of selective attention [17] posits that the allocation of attentional resources depends on the level of perceptual and working memory load. High perceptual load depletes attentional capacity, preventing distractor processing and facilitating early selection. Conversely, low perceptual load leaves spare capacity, allowing for late selection and greater distractor interference.

Liu et al. [22] propose that studies showing decreased fixation durations with increased load may have involved high perceptual load, where fewer items are processed per fixation, resulting in shorter durations and higher fixation rates. Conversely, high working memory load involves processing all relevant items in a single fixation, leading to longer durations and fewer fixations. Therefore, perceptual and working memory load have opposite effects on fixation duration and rate. Liu et al. [22] propose a method to analyze both fixation duration and fixation rate to assess mental load. Their findings indicate that different types of mental load have opposite effects on fixation-related parameters. Specifically, they observed shorter fixation durations and an increased number of fixations under high perceptual load, whereas longer fixation durations and fewer fixations occurred under high working memory load. Thus, perceptual and working memory load exert opposite influences on both fixation duration and fixation rate. To gain deeper insights into sex differences in perception, we combined fixation duration and fixation rate to assess mental load.

For the eye-tracking analysis, saccades were identified using the EyeLink system’s standard parameters and velocity-based saccade detection algorithm. This algorithm calculated instantaneous velocity and acceleration for each data sample and compared these values to preset thresholds. A saccade was detected if either velocity or acceleration surpassed the thresholds, and a fixation was identified once both fell below the thresholds. The system ensured the saccade signal persisted for a critical duration before confirming the initiation or termination of a saccade. The default thresholds for the head-stabilized (chinrest) mode were set at a velocity of 30°/s and an acceleration of 8000°/s^2^. Fixations were recorded whenever the pupil was visible and no saccade was occurring. The data from all participants who completed the experimental protocol were included in the subsequent analyses. There were no exclusions based on data loss, technical issues, or any other criteria.

## 3. Results

### 3.1. Mental Load

**Figure 3:**
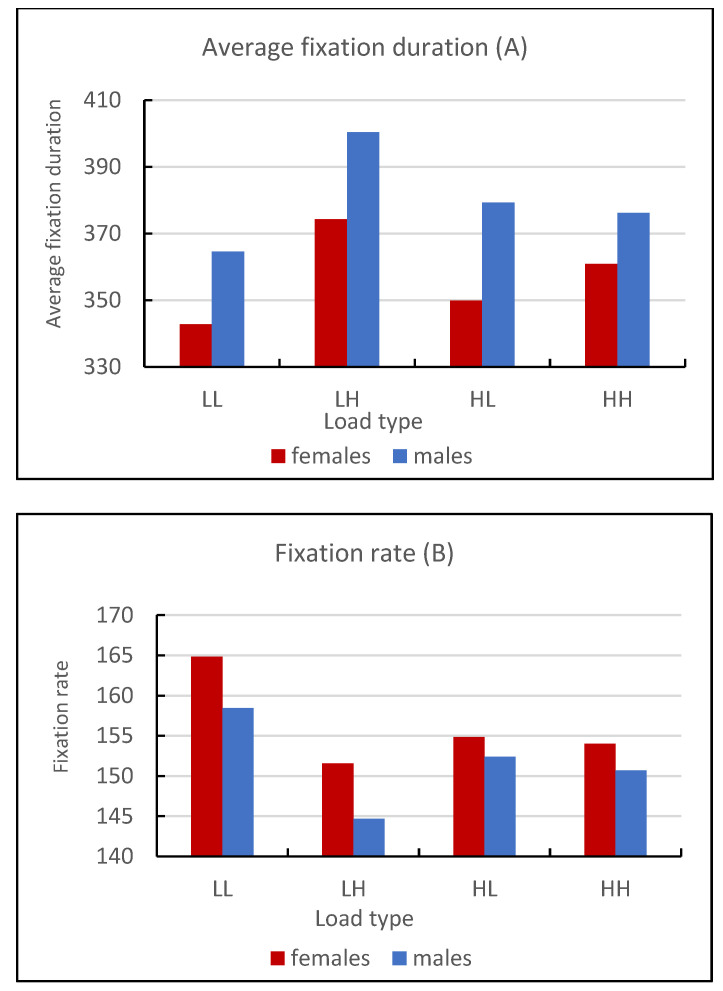
Graphs depicting (**A**) average fixation duration (in ms) and (**B**) fixation rate (fixations per minute) across four conditions of perceptual load (PL) and working memory load (WML): Low–Low (LL: low perceptual load, low working memory load), Low–High (LH: low perceptual load, high working memory load), High–Low: (HL: high perceptual load, low working memory load), and High–High (HH: high perceptual load, high working memory load).

Descriptive statistics for fixation rate and average fixation duration are summarized in Table 1 and Table 2 and Figure 3. The Mann–Whitney U test was used to test the null hypothesis that there are no statistically significant differences in fixation measurements between sex groups. This test was chosen because the data were non-normally distributed, and prior studies recommended using the median for real-world data. Eye movement characteristics were similar to those typically observed in scene-viewing studies.

### 3.2. Average Fixation Duration

A Shapiro–Wilk normality test revealed that the data were non-normally distributed, justifying the use of the Mann–Whitney U test. This test was conducted to investigate the difference in average fixation duration between females (N trials = 264, Mdn = 342.72, IQR = 75.70) and males (N trials = 198, Mdn = 360.42, IQR = 86.64). The results were statistically significant (U = 22,874.00, *p* = 0.022), indicating a difference in average fixation duration between the two groups. Thus, males had significantly higher average fixation durations than females.

### 3.3. Fixation Rate

Using the Mann–Whitney U test, we analyzed the difference in fixation rate between females (Mdn = 157.06, IQR = 32.98) and males (Mdn = 152.8, IQR = 33.65). The data were non-normally distributed. The test results were statistically significant (U = 22,959.50, *p* = 0.025), indicating that females had a significantly higher fixation rate than males. These findings highlight significant differences in fixation measurements between sexes, with females exhibiting shorter fixation durations and higher fixation rates.

### 3.4. Accuracy, Reaction Time, and Numerosity Estimation

The results showed similar accuracy rates for females (56.61%) and males (57.34%) and negligible differences in reaction times (females: 1950.03 ms; males: 1954.8 ms). However, males’ reaction times decreased with task complexity (2300 ms to 1540 ms), suggesting improved efficiency, while females’ reaction times increased (1950 ms to 2200 ms), indicating greater mental effort [18].

The analysis of potential sex differences in numerosity estimation revealed statistically significant differences between males and females (U = 25.000, W = 103.000, *p* = 0.041). On average, females underestimated numerosity more than males (M females = 11.833, SD females = 4.407; M males = 16.333, SD males = 5.292). When comparing estimated numerosity to the actual value (17), males’ estimates (M = −0.667, SD = 5.292) did not significantly differ from the actual numerosity, while females’ estimates (M = −5.167, SD = 4.407) were significantly lower. These findings suggest a potential sex-based bias in numerosity perception, with females tending to underestimate numerosity compared to males [19].

To examine sex differences in eye-tracking measures, we averaged fixation duration and fixation rate across trials for each participant per video, yielding one value per participant per condition. A two-way MANOVA was conducted to examine the effects of sex (male, female) and video (22 levels) on average fixation duration and fixation rate. The results revealed a significant main effect of sex, F (2, 417) = 4.458, *p* = 0.012, indicating overall differences in fixation patterns between males and females. There was also a significant main effect of video, F (42, 836) = 2.261, *p* < 0.001, suggesting that stimulus-specific properties (e.g., object density, target presence) influenced both fixation duration and fixation rate. This video effect was explored to provide context for interpreting sex differences, as variations in scene complexity could modulate eye movement patterns. We did not collapse data by load type due to variability in object counts within categories, and preliminary analyses showed introduced noise.

Follow-up univariate analyses confirmed that sex had a significant effect on average fixation duration, F (1, 418) = 6.663, *p* = 0.010, with females exhibiting shorter fixation durations than males. The effect of sex on fixation rate was marginally significant, F (1, 418) = 3.295, *p* = 0.070, with females tending to make more frequent fixations. The main effect of video remained significant for both average fixation duration, F (21, 418) = 2.204, *p* = 0.002, and fixation rate, F (21, 418) = 3.029, *p* < 0.001, reinforcing the role of stimulus properties in guiding eye movements. The sex × video interaction remained non-significant for both dependent variables (*p* > 0.78).

Females showed shorter fixation durations (Mdn = 342.72 ms) and higher fixation rates (Mdn = 157.06 fixations/min) than males (Mdn = 360.42 ms and 152.80 fixations/min), patterns that align with the load theory of selective attention [17]. These suggest higher perceptual load in females and higher working memory load in males, influencing distractor processing differently across sexes.

## 4. Discussion

Our study builds on Krueger’s pioneering work [4], offering a more nuanced understanding of the factors influencing sex differences in numerosity perception. Krueger suggested that the underestimation of numerosity in females could be due to potential differences in visual acuity, visuospatial processing, and mathematical skills. However, our findings, showing equal performance of females and males in dynamic visuospatial tasks, challenge the notion that these abilities underlie the underestimation in females. Instead, our results indicate that differences in inhibition of distractors between sexes play a significant role. Our proposed explanation rests on the idea that sex differences in numerosity perception become most pronounced in situations requiring selective attention and the inhibition of irrelevant stimuli. Where “distractors” are present, the efficiency of distractor inhibition determines how many items ultimately reach working memory for counting. From our data, it appears that women’s stronger inhibition of distractors causes them to count fewer items, thus systematically underestimating numerosity. Baseline underestimation appears in simpler enumeration tasks (as in Krueger [4]), and additional distractors can accentuate that difference. Rather than contradicting Krueger’s findings, our work offers a mechanism—distractor inhibition—to explain how a pre-existing female bias toward underestimation might be magnified under higher-load conditions.

Our findings are further supported by evidence that numerosity perception is influenced by inhibitory control, as demonstrated across developmental stages. Gray and Reeve [34] noted that general cognitive functions, particularly working memory and response inhibition, support the early development of numerosity estimation (dot enumeration), with research linking these abilities in older children and adults to variations in inhibitory control [35,36,37,38]. Specifically, inhibitory control enhances estimation efficiency by preventing revisits to already processed items [38], a mechanism mirrored in our task where females’ shorter fixation durations and higher fixation rates suggest effective distractor suppression, leading to lower numerosity. Males’ longer fixations and lower rates indicate broader processing, consistent with less efficient inhibition. This aligns with the load theory of selective attention [17], where high perceptual load (PL) in females reduces distractor interference early (p. 342), while males’ higher working memory load (WML) increases it (p. 351).

The observed sex difference in the distribution of mental load suggests that men had to work harder to inhibit the distractors (higher working memory load), but on the other hand, they used fewer resources to inspect the objects (lower perceptual load). High working memory load, due to the inability to suppress irrelevant items, increases the number of items processed within a fixation, thereby lengthening fixation duration and allowing participants to obtain the whole image with fewer fixations [22]. Women, on the other hand, were able to filter out some distractors more effectively, as reflected in their reporting of lower numbers, but they dedicated more attention to inspecting the objects, indicating a higher perceptual load.

Consequently, one potential explanation for sex differences in numerosity perception could be disparities in inhibitory control, which our data suggest modulate load distribution. Research consistently shows sex differences in inhibition, with women demonstrating superior behavioral inhibitory control, evidenced by faster detection and resolution of response conflicts [9]. Although some differences in sustained attentional control, such as omission and commission errors, are tied to sociocultural factors, sex differences in reaction time persist even after accounting for these influences [14]. Given inhibitory control’s critical role in selective attention [17], it likely contributes to our observed sex differences, where females’ efficient distractor rejection aligns with lower estimates and higher PL, while males’ broader processing reflects greater WML. This supports our hypothesis that women’s stronger inhibition leads to a different mental workload distribution compared to men, amplifying underestimation in distractor-rich contexts like our 3D video task.

Displays that create a high degree of competition among objects necessitate a strong bias to select the target for further processing. Several studies indicate that fixation duration tends to increase with increased competition among objects. In tasks where fixation duration increased, it was associated with a rise in the number of inspected items [39]. In our study, objects competed intensely with candidate targets in the video, resulting in high perceptual load. Conversely, minimal competition between task-relevant stimuli requires little top-down control to resolve the conflict, leading to low perceptual load [40]. Torralbo and Beck [41] also found that stimuli inducing greater competition in the visual cortex reduced distractor interference, similar to high perceptual load. Therefore, differences in performance between females and males on visuospatial tasks across various studies may be attributed to differing experimental conditions and the perceptual load experienced by participants. For instance, studies often report mixed results. One study found no significant differences between sexes in the efficiency and accuracy of spatial orientation [6]. In contrast, another study showed that males outperformed females in both large-scale and small-scale spatial abilities, with a significantly larger effect size in large-scale spatial ability [7]. Additionally, a male advantage in spatial processing ability was reported, indicating that males outperformed female students in mental rotation tasks [8].

Interestingly, the findings of this study, which suggest that sex differences in numerosity perception may stem from differences in distractor inhibition and perceptual load distribution, open avenues for future research into cognitive training and its potential to mitigate these differences. Evidence suggests that perceptual capacity limits are not fixed but can be improved through targeted training. Prior studies have demonstrated that both children and adults who are experienced action video game players exhibit significantly greater resistance to distractors under high perceptual load conditions [40,42,43,44]. Longitudinal research further supports this notion, showing that perceptual training effects persist over time, leading to more efficient attentional control and reduced neural activity during similar tasks [45,46]. However, caution is warranted when interpreting these findings, as some studies argue that the causal relationship between action video game training and cognitive improvements is not as robust as often claimed, with pre-existing individual differences potentially driving observed effects [47,48]. Furthermore, while meta-analyses confirm positive impacts of action video games on attention and perception, concerns about methodological limitations, publication bias, and the need for larger, well-controlled studies highlight the necessity for more rigorous experimental designs [49]. Incorporating eye-tracking measures into training studies could help address these concerns by providing more precise insights into how perceptual and working memory loads evolve with practice, ultimately refining adaptive learning strategies.

## 5. Limitations

It is essential to point out that there is no single measure universally valid for determining mental load across varied scenarios. Physiological responses to mental load are highly scenario-dependent and influenced by numerous task characteristics and individual differences. Consequently, different physiological measures perform differently in various study contexts [24]. In their literature review, Tao et al. reported that fixation duration showed a statistically significant difference in discriminating tasks with varied mental load levels in 73% of cases. Although studies suggest that fixation-related information can reflect the effect of mental load, opposite effects have been found. For example, as mental load increased, fixation duration was either found to increase [31,32] or decrease [22,30]. These apparently contradictory findings may result from imprecise definitions of perceptual load and the assessment of different types of mental load. Specifically, fixation duration decreased as task demand increased in simulated flight tasks [50] and simulated driving tasks [51]. Fixation rate was positively correlated with mental load in pilot mission tasks [52] and hypermedia interaction tasks [53]. An important limitation of this study is its relatively small sample size. While our findings suggest a consistent pattern of results, caution should be taken when generalizing them to larger populations. Future research with a more substantial participant pool would help confirm the robustness of these effects and enable more comprehensive statistical modeling. Furthermore, the uneven distribution of trial types, designed to mirror the real-world variability of the KITTI dataset, might have subtly shaped the observed load effects across conditions, enriching our understanding of their dynamic interplay.

## 6. Conclusions

In our experiment measuring both visuospatial abilities and numerosity estimation, we found that females and males had comparable reaction times and accuracy. However, females consistently underestimated numerosity. This underestimation was linked to lower working memory load and higher perceptual load in females, as evidenced by shorter fixation durations and higher fixation rates. Taken together, this suggest that women are better at inhibiting distractors and, consequently, report lower and less accurate numerosity estimates compared to men, who are less efficient at inhibiting the distractors.

The similarity in reaction times and accuracy suggests that basic visuospatial abilities are comparable between sexes. However, the observed difference in the distribution of mental load between working memory load and perceptual load implies that there is a difference in strategies men and women employ in visual perception tasks. This does not necessarily point to differences in underlying visuospatial abilities but rather indicates that a difference in how the inhibitory mechanisms function affects how these abilities are utilized.

Our study addresses a previously underexplored aspect of numerosity estimation by examining sex differences in both numerosity estimation and visuospatial processing. These findings contribute to a deeper understanding of numerosity perception across different contexts and populations and suggest promising directions for future research.

Understanding how perceptual load influences numerosity estimation can inform educational strategies and cognitive training programs designed to mitigate these differences. Future studies should continue to explore these factors to enhance our comprehension of numerosity perception, ultimately contributing to more effective interventions and support systems for diverse populations.

## Figures and Tables

**Table 1:** Mann–Whitney U test results.

Variable	Mann–Whitney U Test by Variable Sex. Marked Tests are Significant at *p* < 0.05						
	Rank Sum Females	Rank Sum Males	U	Z	*p*-Value	Valid N Females	Valid N Males
Average fixation duration	57,854.0	49,099.0	22,874.0	−2.3	0.022	264	198
Fixation rate (per minute)	64,292.5	42,660.5	22,959.5	−2.2	0.025	264	198

**Table 2:** Aggregate results’ descriptive statistics.

Variable	Aggregate Results’ Descriptive Statistics								
	Sex	N trials	Mean	Median	Minimum	Maximum	Variance	Std.Dev.	Coef.Var.
Average fixation duration	Females	264	357.36	342.72	226.23	788.11	5391.19	73.42	20.55
Fixation rate (per minute)	Females	264	156.59	157.06	74.83	235.02	637.99	25.26	16.13
Average fixation duration	Males	198	377.69	360.42	190.67	804.37	9895.53	99.48	26.34
Fixation rate (per minute)	Males	198	151.88	152.80	70.43	272.73	1065.22	32.64	21.49

## Data Availability

Data are contained within this article.
